# Zinc oxide nanoparticles enable sustainable disease management in tea by dual nutrient and antifungal action

**DOI:** 10.1515/biol-2025-1260

**Published:** 2026-02-03

**Authors:** Debajit Saikia, Pradip Kumar Baruah, Satya Ranjan Sarmah, Ram Prasad, Hemen Sarma

**Affiliations:** Department of Botany, University of Science and Technology, Ri-Bhoi, Techno City, Killing Road, 793101 Baridua, Meghalaya, India; Department of Botany, Chandra Nath Bezbaruah College, Bokakhat, 785612 Golaghat, Assam, India; Department of Mycology and Microbiology, Tocklai Tea Research Institute, Cinnamara, 785008 Jorhat, Assam, India; Department of Botany, Mahatma Gandhi Central University, 845401 Motihari, Bihar, India; Department of Botany, Bodoland University, Rangalikhata, 783370 Deborgaon, Assam, India

**Keywords:** zinc oxide nanoparticles, *Camellia sinensis*, *Fusarium solani*, zinc uptake, microbial biomass carbon

## Abstract

Zinc oxide nanoparticles (ZnO-NPs) are emerging as effective micronutrient carriers with additional antifungal properties. However, their application in perennial plantation crops such as tea (*Camellia sinensis*) remains unexplored. *Fusarium solani*, a destructive soil-borne pathogen, poses a significant challenge in tea nurseries and plantations. Greenhouse pot trials were conducted using ZnO-NPs at 3, 6, and 9 mg kg^−1^, with ZnSO_4_·7H_2_O serving as the conventional zinc control. Disease severity, rhizosphere colony-forming units (CFU) populations, soil zinc availability, foliar uptake, microbial biomass, and chlorophyll traits were assessed over 30 days. ZnO-NPs reduced disease severity by 18–55 % and suppressed rhizosphere *F. solani* CFU counts by up to 69 %, significantly outperforming ZnSO_4_·7H_2_O. They increased DTPA-extractable soil Zn (to 0.84 mg kg^−1^) and improved foliar Zn uptake. In comparison, the 6 mg kg^−1^ dose enhanced chlorophyll *a* and total chlorophyll, while maintaining near-baseline microbial biomass. Although the 9 mg kg^−1^ dose yielded higher pathogen suppression, it reduced microbial biomass carbon by 19 %. Microscopy confirmed collapsed hyphae and deformed conidia, consistent with oxidative stress and cell wall disruption. This study provides the first greenhouse-based evidence that ZnO-NPs can function as dual-action soil amendments in tea, improving both nutrient status and resistance to *F. solani*. The intermediate rate (6 mg kg^−1^) delivered the best balance between plant benefits and microbial stability, highlighting the agronomic promise of nano-enabled inputs. Further multi-season field studies are needed to verify their effectiveness and environmental safety.

## 1 Introduction

Tea (*Camellia sinensis* L.) is one of India’s most crucial plantation crops, contributing significantly to both rural livelihoods and national exports. However, its long-term productivity is increasingly constrained by soil nutrient depletion and soil-borne pathogens. Among essential micronutrients, zinc (Zn) is ranked just after nitrogen, phosphorus, and potassium in limiting crop productivity [1]. Nearly 50 % of Indian soils are Zn-deficient, with surveys in Assam consistently reporting extractable Zn levels below the sufficiency threshold of 0.6 mg kg^−1^ [2], 3]. As Zn plays pivotal roles in photosynthesis, enzyme activation, and oxidative stress regulation its deficiency directly compromises the growth and resilience of tea plants [4], 5].

Simultaneously, soil-borne fungal diseases, particularly dieback caused by *Fusarium solani*, represent a major pathological constraint. The pathogen survives in soil through persistent chlamydospores, spreads via irrigation and planting materials, and causes dieback symptoms that can reduce productivity by up to 40–50 % [6], 7]. Chemical fungicides provide only partial suppression, raising concerns about environmental toxicity, non-target impacts, and resistance development [8], 9]. Hence, there is an urgent need for novel antifungal strategies that combine efficacy with ecological safety.

Nanotechnology has emerged as a frontier in pesticide biochemistry and plant protection, offering materials with enhanced surface reactivity, controlled ion release, and direct antimicrobial interactions. Zinc oxide nanoparticles (ZnO-NPs) are particularly promising due to their dual functionality: sustained Zn^2+^ release to correct micronutrient deficiencies, and intrinsic nano-fungicidal activity. Studies in vegetable and legume crops demonstrate that ZnO-NPs suppress *Fusarium* wilt and root rot while improving plant nutrition and physiological traits [10], [11], [12]. Reviews consistently identify ZnO-NPs as among the most potent nano-enabled antifungal agents, acting through mechanisms such as cell wall disruption, oxidative stress induction, and metabolic inhibition [13], [14], [15].

Despite these promising findings, no systematic study has investigated whether ZnO-NPs can simultaneously manage *F. solani* dieback and improve Zn nutrition in tea, a perennial plantation crop of global economic importance. To address this gap, the present study integrates *in vitro* antifungal assays with greenhouse pot experiments to evaluate ZnO-NPs as dual-function soil amendments in tea. The study specifically examined (i) ZnO-NPs-mediated suppression of *F. solani* inoculum and disease severity, (ii) biochemical and physiological responses of tea plants, including Zn uptake and chlorophyll content, (iii) soil chemical dynamics (pH, EC, and extractable Zn), and (iv) soil microbial biomass carbon (MBC) as an ecological safety indicator.

By linking antifungal activity with plant physiological benefits, this work provides, to our knowledge, the first greenhouse pot-based evidence that ZnO-NPs may serve as soil-applied nano-fungicidal amendments for tea. The findings also underscore the need for multi-season field validation to assess long-term agronomic and ecological outcomes.

## 2 Materials and methods

### 2.1 Experimental site and soil collection

The study was conducted at Diffloo Tea Estate, Assam, India (26°36′33″N, 93°35′14″E), a site with a history of *Fusarium* dieback. Topsoil (0–20 cm) was composited from 10 cores, air-dried, sieved (2 mm), and double-sterilized by autoclaving for pot assays. Sterile soil was used in pathogen assays; non-sterile soil was reserved for microbial biomass experiments.

Baseline soil characterization confirmed acidic, sandy loam soil typical of tea-growing regions (Table 1). All pots were filled with the same homogenized soil batch, ensuring uniform starting conditions. Baseline analyses indicated an acidic reaction (pH 5.47 ± 0.03), moderate EC (2.02 ± 0.01 dS m^−1^), low organic carbon (0.59 ± 0.02 %), and low total *N* (0.08 ± 0.01 %). Bulk density (1.52 ± 0.04 g cm^−3^) and porosity (39.9 ± 1.01 %) were consistent with sandy loam soils (Supplementary Method 1). Of particular note, the soil was Zn-deficient (DTPA-Zn 0.39 ± 0.02 mg kg^−1^), well below the agronomic sufficiency threshold (0.6 mg kg^−1^), providing a strong rationale for evaluating ZnO nanoparticles as a Zn source.

**Table 1:** Baseline physicochemical properties of the experimental soil.

Property	Value (mean ± SE, *n* = 3)
Texture (sand/silt/clay, %)	64.5/20.7/14.8
Textural class	Sandy loam
pH (1:5 soil–water)	5.47 ± 0.03
Electrical conductivity (dS m^−1^)	2.02 ± 0.01
Organic carbon (%)	0.59 ± 0.02
Total nitrogen (%)	0.08 ± 0.01
DTPA-extractable Zn (mg kg^−1^)	0.39 ± 0.02
Porosity (%)	39.9 ± 1.01
Bulk density (g cm^−3^)	1.52 ± 0.04

### 2.2 Zinc oxide nanoparticle characterization and dispersion

Commercial ZnO nanoparticles (nominal size <30 nm, 99 % purity; Ultrananotech, Bangalore, India) were dispersed in deionized water with 0.01 % Tween-80 and probe-sonicated (100 W, 20 kHz, 10 min) to ensure a stable suspension. Nanoparticles were characterized using standard techniques: X-ray diffraction (XRD) for crystal structure, Fourier transform infrared spectroscopy (FTIR) for surface functional groups, field emission scanning electron microscopy (FESEM) with energy-dispersive X-ray spectroscopy (EDS) for morphology and composition, and *ζ*-potential analysis for colloidal stability (Supplementary Method 2).

### 2.3 Pathogen isolation, inoculum preparation, and molecular identification

**Figure 1:**
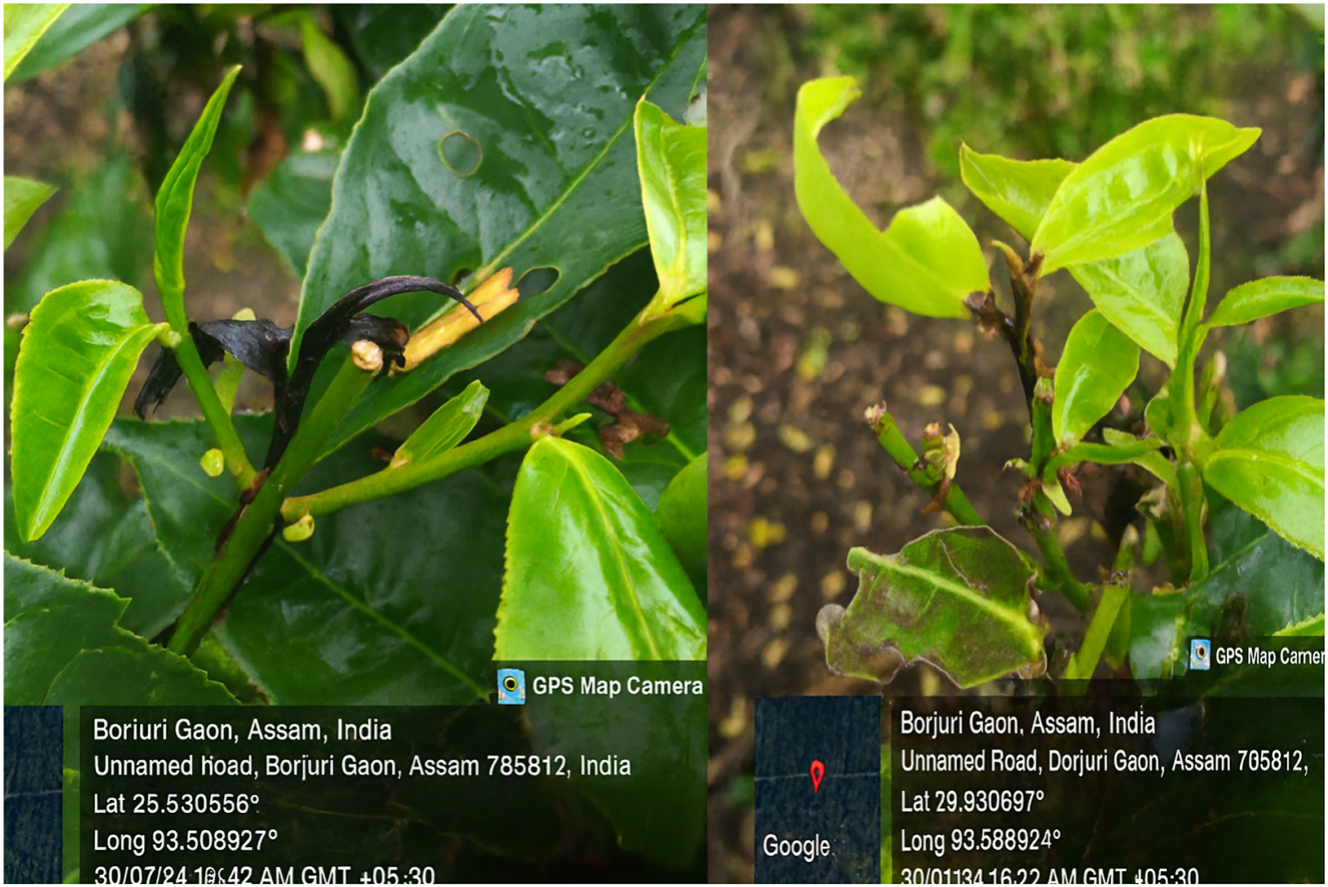
Tea plants showing Fusarium dieback symptoms in Diffloo Tea Estate, Assam, India. Representative field photographs illustrate typical shoot dieback symptoms caused by *Fusarium solani*.

Symptomatic tea shoots showing dieback (Figure 1) were collected from Diffloo Tea Estate, Assam, India (26°37′50″N, 93°34′08″E). *F. solani* was isolated on potato dextrose agar (PDA) and identified based on colony morphology and internal transcribed spacer (ITS) region sequencing. Pure cultures were maintained on PDA at 25 ± 1 °C.

For inoculum preparation, seven-day-old cultures were flooded with sterile distilled water and filtered through muslin cloth. The conidial suspension was standardized to 1 × 10^5^ conidia mL^−1^ using a hemocytometer.

For molecular identification, genomic DNA was extracted from fresh mycelium using a CTAB–phenol–chloroform method. The ITS region of rDNA was amplified with primers ITS1 and ITS4, sequenced, and deposited in GenBank (Accession PV171106.1). BLASTN confirmed species identity. Phylogenetic placement was assessed using Maximum Likelihood in MEGA v12 with 1,000 bootstrap replicates and representative *Fusarium* spp. Plus tea pathogens as outgroups. The isolate clustered within the *F. solani* clade with strong bootstrap support.

### 2.4 Pot experiment design, treatments, and inoculation

Greenhouse trials were conducted under controlled conditions (30 ± 3 °C, 65–75 % relative humidity, 14 h light/10 h dark). Soil moisture was maintained near field capacity by regular watering, and separate trays and tools were used for each treatment to avoid cross-contamination. The experiment followed a completely randomized design (CRD). Each pot (8 × 10-inch plastic, 5 kg sterilized sandy loam soil) contained a single tea seedling and served as the experimental unit. Each treatment comprised six replicates (*n* = 6), though only three pots per treatment were used for chlorophyll and foliar Zn estimations due to resource limitations.

Six treatments were established (Table S1). The experimental treatments were as follows: T1 (Sterile control); T2 (Inoculated control); T3 (ZnO-NPs 3 mg kg^−1^ + *F. solani*); T4 (ZnO-NPs 6 mg kg^−1^ + *F. solani*); T5 (ZnO-NPs 9 mg kg^−1^ + *F. solani*); and T6 (ZnSO_4_·7H_2_O at a zinc dose equivalent to T5 + *F. solani*).

At transplanting (Day 0), inoculated pots received 100 mL of a standardized *F. solani* suspension (1 × 10^5^ conidia mL^−1^; ≈1 × 10^7^ conidia pot^−1^). Negative controls received sterile water. ZnO-NPs were suspended in deionized water with 0.01 % Tween-80, probe-sonicated, and applied as a single soil drench (100 mL per pot).

Notes: T1 excluded from DSI because uninoculated, T2 excluded from MBC assays and *in vitro* EC_50_ against *F. solani* ≈ 310 mg L^−1^ (Supplementary Table S2), used to select soil application rates.

### 2.5 Sampling strategy and measurements

Plant and soil samples were collected at 7, 14, and 30 days after transplanting. Soil measurements, including pH, electrical conductivity (EC), DTPA-extractable Zn, and microbial biomass carbon (MBC), were taken with six replicates (*n* = 6). Plant response variables such as disease severity index (DSI) and *F. solani* CFU counts were also assessed with six replicates (*n* = 6). In comparison, chlorophyll content and foliar Zn concentrations were measured with three replicates (*n* = 3). Rhizosphere soil (0–10 cm) was sieved through a 2-mm mesh and processed immediately. Fungal colonies were quantified using Nash–Snyder selective medium supplemented with streptomycin (50 mg L^−1^) after incubation at 27 ± 2 °C for 5 days, with representative colonies confirmed microscopically and by ITS sequencing (Supplementary Method 3; Table S3).

### 2.6 Plant measurements and response variables

#### 2.6.1 Disease severity index (DSI)

Disease severity was evaluated on all leaves of each seedling at 14- and 30-day post-inoculation using a 0–5 scale (Table S3) adapted from Sarmah et al. [16]. For each pot, leaf scores were converted to a pot-level DSI (%) using the formula:

$$\text{DSI}\%=\frac{\sum \left(\text{class}\;\text{score}\times \text{number}\;\text{of}\;\text{leaves}\right)}{\max\;\text{score}\times \text{total}\;\text{leaves}}\times 100$$

#### 2.6.2 Phytotoxicity screening

Seedlings were monitored up to 30 days after the first inoculation for possible phytotoxic effects, including tip burn, surface lesions, wilting, necrosis, vein clearing, epinasty, and hyponasty. The severity of these symptoms was quantified using a percentage-based grading system (Table S4), which classified phytotoxicity into 10 levels ranging from 0 (no visible effect) to 10 (91–100 % injury).

#### 2.6.3 Physiological measurements (chlorophyll and Zn uptake)

Chlorophyll content and foliar Zn concentration were used as physiological indicators of plant response. Chlorophyll was determined at 14 and 30 days from fully expanded upper-canopy leaves (*n* = 3 per treatment). Approximately 0.2 g of fresh tissue was extracted in ethanol, and absorbance was recorded at 665 and 649 nm using a Shimadzu UV–2600i spectrophotometer (Japan). Concentrations of chlorophyll *a*, *b*, and total chlorophyll were calculated using Lichtenthaler and Wellburn [17]: Chlorophyll *a* (mg g^−1^ FW) = 13.95 × A_665_ − 6.88 × A_649_

$$\text{Chlorophyll }b\;\left(\text{mg }g^{-1}\text{ FW}\right)=24.96\times A_{649}–7.32\times A_{665}$$

$$\text{Total chlorophyll }\left(\text{mg }g^{-1}\text{FW}\right)=\text{Chl}a+\text{Chl}b$$

Values were expressed on a fresh weight basis (mg g^−1^ FW).

Leaf Zn was measured at 0, 7, 14, and 30 days after maturity from fully expanded leaves (*n* = 3 per treatment). Samples were oven-dried, ground, and digested in nitric–perchloric acid (3:1, v/v). Zn concentration in digests was quantified by atomic absorption spectrophotometry (PerkinElmer AAnalyst 400) and expressed as mg Zn kg^−1^ dry weight. Details of chlorophyll estimation and Zn analysis in tea seedlings are provided in Supplementary Table S5.

### 2.7 In vitro antifungal bioassays and scanning electron microscopy

A virulent isolate of *F. solani* (DSP-1) was cultured in potato dextrose broth at 25 ± 2 °C (120 rpm, 24 h) to obtain actively growing hyphae and conidia. Fresh ZnO-NP suspensions were probe-sonicated in 0.01 % Tween-80 and immediately added to cultures. Cultures were exposed to ZnO-NPs (100–1,200 mg L^−1^) and radial growth was measured after 5 days. The EC_50_ was estimated at ∼310 mg L^−1^ by probit analysis (Table S2). Based on this, three concentrations (150, 300, 450 mg L^−1^) were selected for detailed assays and ultrastructural observations.

Mycelia and conidia from treated and control plates were harvested after 5 days, fixed, dehydrated, gold-coated, and examined under a JEOL JSM-6390LV scanning electron microscope. Treated hyphae showed surface disruption compared with smooth control hyphae. Morphometric measurements of *F. solani* under ZnO-NP treatments are provided in Supplementary Table S6.

### 2.8 Auxiliary non-sterile pot experiment: microbial biomass carbon under ZnO-NP exposure

To evaluate the ecological impact of ZnO-NPs on soil microbial activity, an auxiliary pot experiment was conducted under non-sterile greenhouse conditions using field soil. A completely randomized design (CRD) was applied with five treatments as described in Section 2.4.1. Each treatment was replicated following the method described in Section 2.5. Microbial biomass carbon was estimated by the chloroform fumigation–extraction method. Extractable organic C in the fumigated and non-fumigated extracts was determined by dichromate oxidation followed by titration against ferrous ammonium sulfate. MBC was calculated as [18]:

$$\text{MBC}=\frac{C_{\text{fum}}-C_{\text{unfum}}}{k_{EC}}$$

Where:

– *C*_fum_ = extractable organic *C* from fumigated soil (mg C kg^−1^)
– *C*_unfum_ = extractable organic *C* from unfumigated soil (mg C kg^−1^)
– *k*_*EC*_ = extraction efficiency coefficient, taken as 0.45

Values were expressed on an oven-dry soil basis.

### 2.9 Statistics

All experiments followed a completely randomized design (CRD). Treatment means were expressed as mean ± SE (*n* = 6, except chlorophyll and foliar Zn, where *n* = 3). Disease severity index, Fusarium CFU, and microbial biomass carbon were analyzed by two-way repeated-measures ANOVA (Treatment × Day), and treatment means were separated by Tukey’s HSD at p ≤ 0.05; effect sizes (ω^2^) were reported only for MBC. Chlorophyll and foliar Zn data were analyzed by two-way ANOVA with ω^2^, and foliar Zn was additionally validated with the Kruskal–Wallis test.

Explicit checks of the underlying statistical assumptions were conducted for all ANOVA procedures. Residual normality was evaluated using the Shapiro–Wilk test and Q–Q plots, while homogeneity of variances was examined using Levene’s test. For repeated-measures analyses, sphericity was assessed with Mauchly’s test; however, because each repeated factor contained only two levels, sphericity was inherently met (W = 1.00). Both Greenhouse–Geisser and Huynh–Feldt corrections were reviewed, and in all cases *ε* remained 1.00, indicating that these adjustments did not affect the outcomes.

Analyses were performed in OriginPro 2018; figures were generated in Python v3.10, and SuperPlots were generated using the web-based tool Super Plots Of Data. Phylogenetic tree construction was carried out in MEGA v12.

## 3 Results

### 3.1 Nanoparticle characterization

#### 3.1.1 X-ray diffraction (XRD) analysis

**Figure 2:**
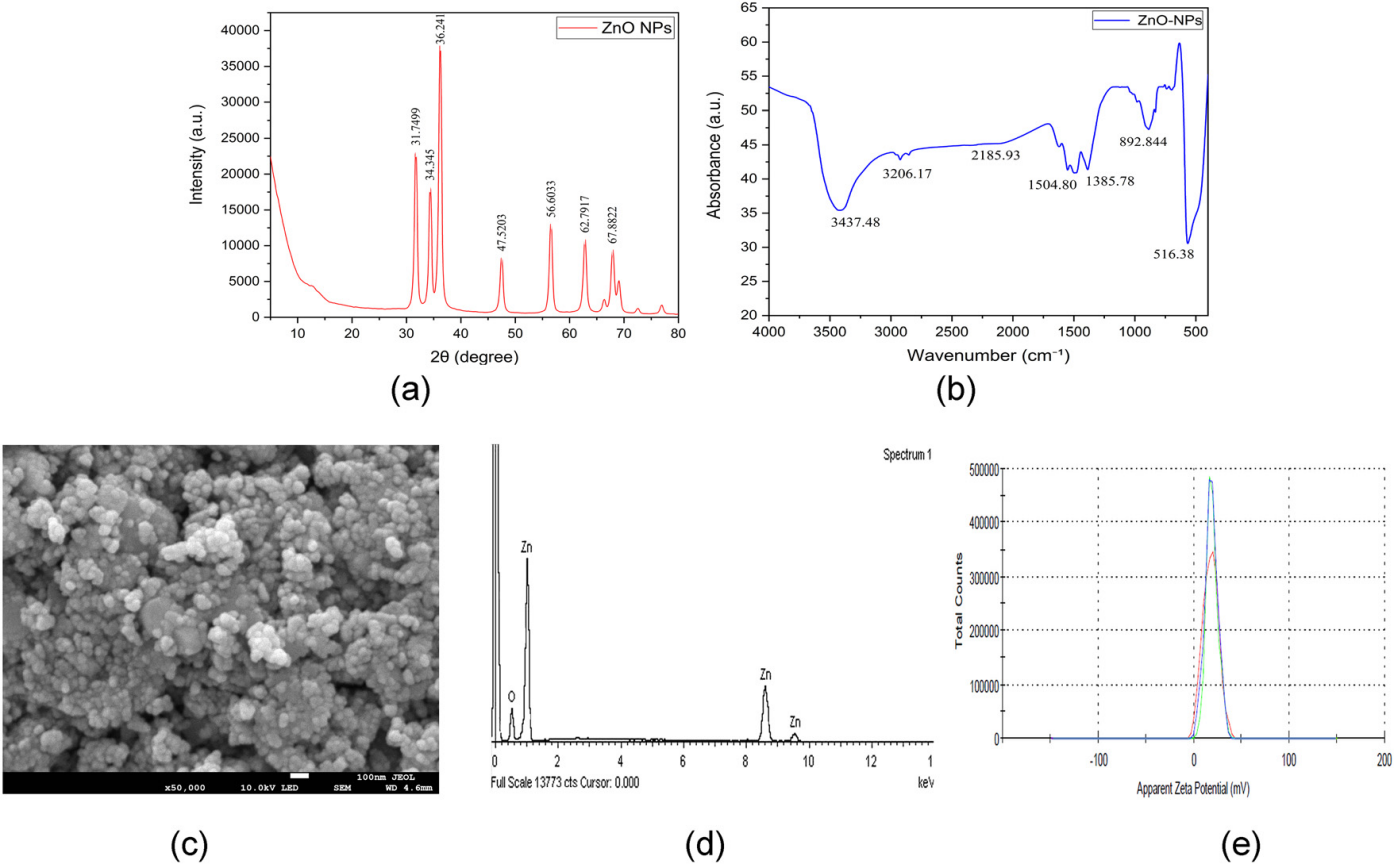
Physicochemical characterization of ZnO nanoparticles. (a) XRD confirming wurtzite phase with ∼13 nm crystallite size. (b) FTIR spectrum showing Zn–O stretching and surface –OH groups. (c) FESEM micrograph (50,000×) showing quasi-spherical nanoparticles (5–25 nm). (d) EDS spectrum confirming Zn and O composition. (e) *ζ*-potential distribution of ZnO-NP suspension at 800 ppm (+18.5 ± 0.8 mV), indicating intermediate colloidal stability.

The XRD pattern of ZnO nanoparticles exhibited sharp peaks at 2θ ≈ 31.7°, 34.4°, 36.2°, 47.5°, 56.0°, 62.8°, and 67.9°, indexed to the (100), (002), (101), (102), (110), (103), and (112) planes of wurtzite ZnO (JCPDS 36–1451), confirming phase purity (Figure 2a). The Scherrer equation estimated an average crystallite size of 13.21 ± 0.18 nm, which was consistent with the Williamson–Hall approach (≈13.25 nm; *ε* ≈ 1.7 × 10^−5^), indicating high crystallinity and low strain. Refined lattice parameters were a = 3.2679 Å and c = 5.1931 Å (c/a = 1.5891), slightly contracted relative to bulk ZnO (a = 3.25 Å, c = 5.20 Å; c/a ≈ 1.60). Such nanoscale deviation, attributed to surface stress and oxygen vacancies, has been previously reported in ZnO nanocrystals <30 nm in size [19], 20].

#### 3.1.2 Fourier transform infrared spectroscopy (FTIR)

The FTIR spectrum of ZnO nanoparticles exhibited distinct absorption bands at 516, 892, 1,346, 1,505, 1,733, 2,250, 2,591, and 3,132 cm^−1^ (Figure 2b), as identified through Gaussian deconvolution (R^2^ = 0.990; χ^2^ = 1.74 × 10^−3^). The strong band at 516 cm^−1^ corresponds to Zn–O stretching, confirming the formation of the ZnO lattice. Weak bands at 892 and 1,346 cm^−1^ were assigned to C–H bending and nitrate-related vibrations, suggesting minor residual precursors. The feature was attributed to the reflected C–H deformation of organic fragments [21]. A broad absorption at 3,132 cm^−1^ was attributed to O–H stretching of surface hydroxyl groups and bound water molecules, which is consistent with the hydroxylated nature of ZnO surfaces [22]. The presence of surface –OH groups is particularly significant, as they enhance dispersion and colloidal stability and contribute to reactive oxygen species (ROS) generation, reinforcing the functional properties of ZnO-NPs reported in earlier studies [23].

#### 3.1.3 Field emission scanning electron microscopy (FESEM) analysis

The FESEM micrograph (×50,000; 5.0 kV) revealed quasi-spherical ZnO nanoparticles with a tendency to agglomerate (Figure 2c). ImageJ-based analysis of 189 particles indicated diameters primarily in the 5–25 nm range, with a mean size of 16.5 ± 2.39 nm. XRD analysis of the same batch exhibited the characteristic wurtzite ZnO pattern (JCPDS 36–1451), with prominent reflections at 2θ ≈ 31.8° (100), 34.4° (002), and 36.3° (101). The average crystallite size estimated by the Scherrer equation (K = 0.9, Cu Kα, background- and instrument-corrected FWHM) was comparable to the FESEM mean, suggesting that most nanoparticles are single-crystal or consist of only a few coherently diffracting domains.

Overall, the close agreement between FESEM particle size and XRD crystallite size confirms the nanoscale dimension of the ZnO nanoparticles with minimal lattice distortion. The observed distribution is consistent with previous reports on ZnO nanomaterials and supports their enhanced surface reactivity, photocatalytic efficiency, and antifungal potential [24].

#### 3.1.4 Energy-dispersive X-ray spectroscopy (EDS)

The EDS spectrum (Figure 2e) shows strong and distinct peaks corresponding to zinc (Zn) at ∼1.0 keV (Zn Lα), ∼8.6 keV (Zn Kα), and ∼9.5 keV (Zn Kβ), along with a clear oxygen (O Kα) peak at ∼0.5 keV. No significant peaks corresponding to other impurities were detected, confirming the sample’s high purity. Quantitative analysis revealed that the sample contained 79.73 wt% Zn (49.06 at.%) and 20.27 wt% O (50.94 at.%), with a total normalized composition of 100 %. The near 1:1 Zn:O atomic ratio indicates the formation of stoichiometric zinc oxide (ZnO).

#### 3.1.5 *ζ*-potential analysis

The 800 ppm ZnO nanoparticle suspension exhibited positive *ζ*-potential values ranging from 17.7 to 19.3 mV, with an average of 18.5 ± 0.8 mV (Figure 2e; Table S7). The corresponding electrophoretic mobility ranged from 1.385 to 1.51 μm cm/Vs, while conductivity values remained stable (∼0.097 mS/cm). The wall *ζ*-potential ranges from −10.4 to −12.0 mV. These results indicate that the nanoparticles possessed a moderately positive surface charge under the experimental conditions, providing a reasonable degree of colloidal stability through electrostatic repulsion.

### 3.2 Molecular identification and phylogenetic analysis

Genomic DNA extracted from the representative isolate DSP-1 yielded a clear, intact band on agarose gel, confirming suitability for downstream analysis. PCR amplification using ITS1/ITS4 primers produced a single ∼550 bp amplicon, consistent with the expected size of fungal ITS regions. The ITS rDNA sequence of *F. solani* isolate DSP-1 (∼593 bp) has been deposited in GenBank under accession number PV171106.1. BLASTN analysis revealed >99 % identity with reference sequences of *F. solani*, thereby validating the morphological diagnosis (Figure S2).

**Figure 3:**
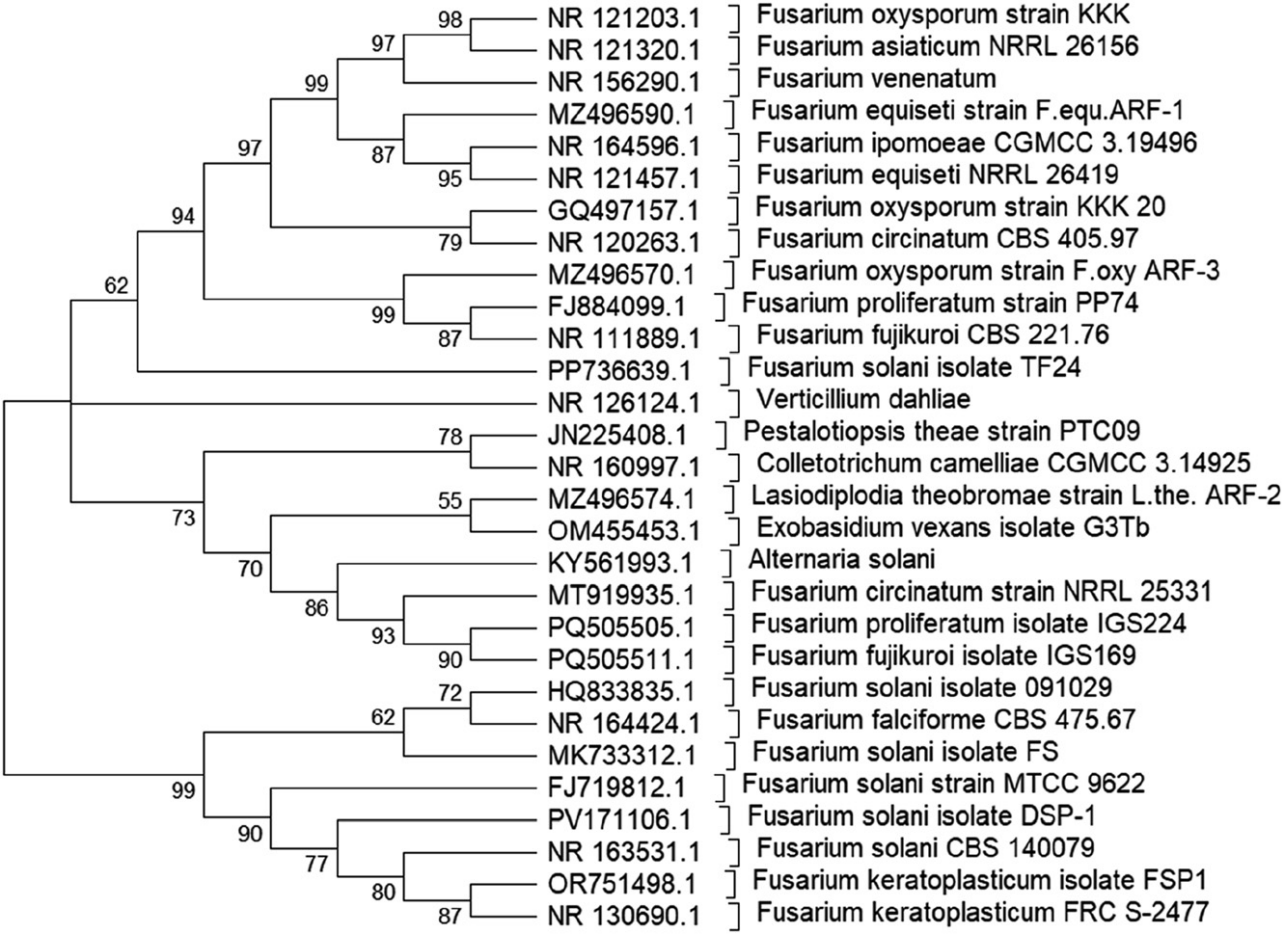
Maximum Likelihood phylogenetic tree of the *Fusarium solani* isolate from tea. The tree was constructed from ITS sequences in MEGA12 using the Maximum Likelihood method with 1,000 bootstrap replicates. Bootstrap values ≥ 50 % are shown at branch nodes. The study isolated PV171106.1 (*Fusarium solani* isolate DSP-1), which clustered within the *F. solani* species complex with strong bootstrap support, confirming its identity as *F. solani*.

The ML phylogenetic tree revealed that the studied isolate clustered within the *F. solani* clade, forming a well-supported branch with reference strains *F. solani* (e.g., NR163531.1, NR164424.1, FJ719812.1, HQ833835.1, and others). This cluster was supported by high bootstrap values (>90 %), indicating strong confidence in the isolate’s placement within the *F. solani* species complex (Figure 3).

### 3.3 Soil measurements and response variables

Soil pH was significantly affected by treatment (F(5,72) = 8.813, p < 0.001, ω^2^ = 0.308), while replicate and interaction effects were not significant. pH values remained within the acidic range favorable for tea (5.43–5.49) and stabilized by Day 30 (5.43–5.46). Post-hoc tests indicated that ZnSO_4_·7H_2_O consistently differed from both controls and ZnO-NP treatments, whereas the ZnO-NPs did not differ significantly among themselves or from the inoculated control.

In contrast, soil electrical conductivity (EC) was not significantly affected by treatment, replicate, or their interaction (F (5,72) = 1.48, p = 0.206, ω^2^ = 0.03). EC values remained stable across treatments and sampling times (2.02–2.06 dS m^−1^), indicating that Zn applications at the tested rates did not alter the soil’s ionic balance.

DTPA-extractable Zn responded strongly to Zn inputs. Baseline availability was low (0.40 ± 0.01 mg kg^−1^), below the sufficiency threshold of 0.6 mg kg^−1^, and remained unchanged in both controls (T1 and T2: 0.39–0.44 mg kg^−1^). In contrast, T3–T6 significantly increased DTPA-Zn in a dose-dependent manner (two-way ANOVA: treatment, Day, and interaction all p < 0.001; Table 2). Overall, T5 outperformed all treatments by maintaining the highest Zn availability over time, consistent with previous incubation studies [25], 26].

**Table 2:** Effect of ZnO nanoparticles and ZnSO_4_·7H_2_O on DTPA-extractable Zn (mg kg^−1^) in soil at 0, 14, and 30 days after treatment. Values are mean ± SE (*n* = 6). ΔZn represents the net change between Day 30 and Day 0. Different superscript letters within a column indicate significant differences at p ≤ 0.05 (Tukey’s HSD). The two-way ANOVA showed highly significant effects of treatment (F(5, 90) = 346.50), Day (F(2, 90) = 20.32), and their interaction (F(10, 90) = 5.99, p < 0.001).

Treatment	Day 0 (mg kg^−1^)	Day 14 (mg kg^−1^)	Day 30 (mg kg^−1^)	ΔZn (30–0)
T1	0.40 ± 0.01^d^	0.41 ± 0.01^e^	0.39 ± 0.01^d^	−0.01
T2	0.42 ± 0.01^c^	0.44 ± 0.01^d^	0.41 ± 0.01^d^	−0.01
T3	0.46 ± 0.01^c^	0.49 ± 0.01^d^	0.47 ± 0.01^c^	+0.01
T4	0.54 ± 0.02^b^	0.60 ± 0.02^c^	0.65 ± 0.02^b^	+0.11
T5	0.69 ± 0.01^a^	0.77 ± 0.01^a^	0.84 ± 0.01^a^	+0.15
T6	0.66 ± 0.02^a^	0.69 ± 0.02^b^	0.70 ± 0.02^b^	+0.04

**Figure 4:**
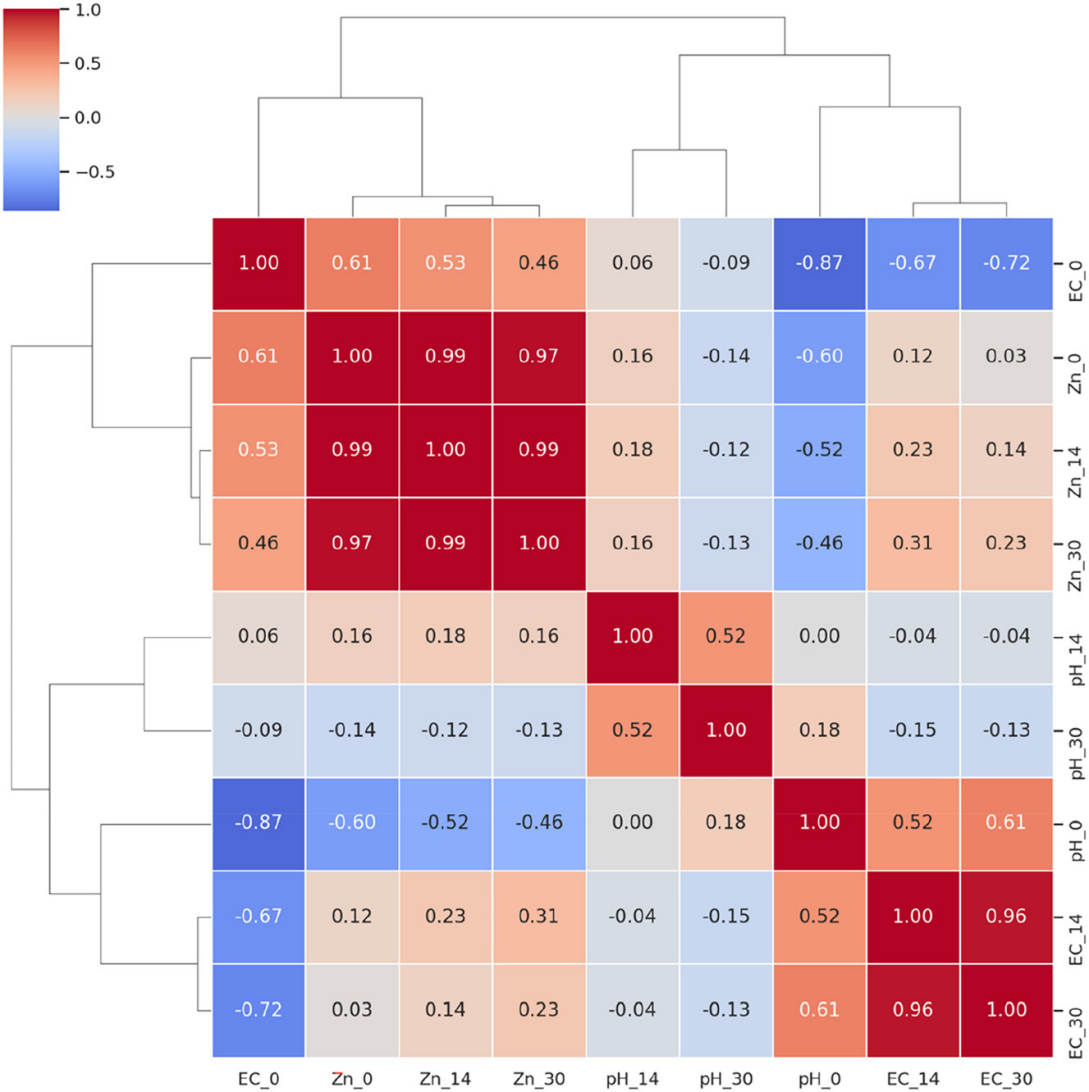
Clustered correlogram of Pearson correlations among soil parameters. Soil pH, electrical conductivity (EC), and DTPA-extractable Zn were assessed at 0, 14, and 30 days under different treatments. Red shades indicate positive correlations, and blue shades indicate negative correlations, with color intensity proportional to the strength of the correlation.

The clustered correlogram (Figure 4) showed that DTPA-Zn at 0, 14, and 30 days grouped into a single, highly coherent cluster (r = 0.97–0.99), confirming stable, treatment-driven Zn dynamics. Zn was moderately negatively correlated with initial pH (r = −0.46 to −0.60), whereas correlations with later pH values were weak, indicating that pH affected Zn mobility only immediately after application. pH variables did not cluster reliably (r = 0.00–0.52). EC showed a similar pattern: EC_14_ and EC_30_ were strongly correlated (r = 0.96), while EC_0_ correlated negatively with both later readings (r = −0.67 to −0.72). Zn showed moderate correlations with EC_0_ (r = 0.46–0.61) but weak associations with EC_14_ and EC_30_ (|r| ≤ 0.23). Overall, only Zn variables formed a stable, treatment-responsive cluster, whereas pH and EC varied independently over time.

### 3.4 *Fusarium* density (CFU g^−1^)

Analysis of rhizosphere CFU counts revealed strong treatment-dependent suppression of *F. solani* populations by ZnO nanoparticles (ZnO-NPs) and ZnSO_4_·7H_2_O (Table 3). A two-way ANOVA showed a significant Treatment effect (F(4,50) = 86.50, p < 0.001), no effect of Day (F(1,50) = 0.007, p = 0.934), and a significant Treatment × Day interaction (F(4,50) = 7.05, p < 0.001). Across both sampling times, the inoculated control maintained the highest CFU densities (2.84 → 3.50 × 10^5^ g^−1^; +23.1 %), while ZnO-NP treatments suppressed fungal populations in a clear dose-dependent manner. Tukey’s HSD confirmed significant reductions in CFU under all ZnO-NP doses (T3–T5) and ZnSO_4_ (T6) relative to T2 (p < 0.01). The lowest dose provided marginal suppression, whereas the intermediate and high doses produced the strongest inhibition at 52.16 % and 69.29 %, respectively. ZnSO_4_·7H_2_O also reduced CFU (20.77 % at Day 14; 47.14 % at Day 30) but was less effective than the highest nanoparticle dose (T5). Overall, ZnO-NPs outperformed ZnSO_4_, with inhibition more pronounced by Day 30.

**Table 3:** *Fusarium solani* population (CFU × 10^5^ g^−1^ oven-dry soil) in rhizosphere soil under ZnO nanoparticle (ZnO-NPs) and ZnSO_4_·7H_2_O treatments at 14 and 30 days after inoculation. Values are means ± SE (*n* = 6). Different superscript letters within a column indicate significant differences according to Tukey’s HSD (p ≤ 0.05). Two-way ANOVA: Treatment F(4,50) = 86.50, p < 0.0001; Day F(1,50) = 0.007, p = 0.934 (ns); Treatment × Day F(4,50) = 7.05, p < 0.001.

Treatment	Day 14 (Mean ± SE)	Day 14 suppression %	Day 30 (Mean ± SE)	Day 30 suppression %
T2	2.84 ± 0.15^a^	0.0	3.50 ± 0.17^a^	0.0
T3	2.15 ± 0.11^b^	24.62	2.34 ± 0.13^b^	33.24
T4	1.63 ± 0.10^b^	42.81	1.50 ± 0.10^b^	57.14
T5	1.36 ± 0.08^c^	52.16	1.07 ± 0.07^c^	69.29
T6	2.25 ± 0.11^c^	20.77	1.85 ± 0.09^c^	47.14

### 3.5 Plant measurements and response variables

#### 3.5.1 Disease severity index (DSI%)

Application of ZnO nanoparticles and ZnSO_4_·7H_2_O significantly reduced *F. solani*–associated disease severity compared with the inoculated control (T2) (Table 4). Two-way ANOVA showed strong main effects of treatment (F(4,50) = 481.44, p < 0.0001, ω^2^ = 0.972) and day (F(1,50) = 1,277.29, p < 0.0001, ω^2^ = 0.961), along with a significant treatment × day interaction (F(4,50) = 83.45, p < 0.0001, ω^2^ = 0.857). Disease severity increased from 14 to 30 dpi across all treatments. At 14 dpi, suppression followed the order T5 > T4 > T6 > T3. By 30 dpi, effects increased, with T5 maintaining the highest protection (55.3 %) and T4 remaining effective (40.3 %), while T3 and T6 provided only moderate reductions (∼18 %). Tukey’s HSD consistently identified T5 as having the lowest DSI and T2 as the highest. At 30 dpi, T3 and T6 formed a statistically similar group; T4 occupied an intermediate position; and T5 remained distinctly superior. Overall, these results demonstrate that ZnO-NPs, particularly at 6–9 mg kg^−1^, confer more substantial and more sustained protection against *F. solani* than conventional ZnSO_4_·7H_2_O.

**Table 4:** Disease severity index (DSI, %) of tea seedlings inoculated with *Fusarium solani*. Values are means ± SE (*n* = 6). Percent suppression was calculated relative to the inoculated control (T2). Different superscript letters within each column indicate significant differences according to Tukey’s HSD (p ≤ 0.05). Two-way ANOVA revealed highly significant effects of treatment (F(4,50) = 481.44, p < 0.0001, ω^2^ = 0.972), day (F(1,50) = 1,277.29, p < 0.0001, ω^2^ = 0.961), and the treatment × day interaction (F(4,50) = 83.45, p < 0.0001, ω^2^ = 0.857).

Treatment	Mean ± SE (14 dpi)	Suppression (%)	Mean ± SE (30 dpi)	Suppression (%)
T2	44.96 ± 0.91^a^	0.0	74.22 ± 0.81^a^	0.0
T3	42.06 ± 0.61^b^	6.45	60.73 ± 1.21^b^	18.18
T4	34.89 ± 0.45^c^	22.4	44.32 ± 1.11^c^	40.29
T5	27.93 ± 0.60^d^	37.88	33.17 ± 0.58^d^	55.31
T6	40.61 ± 0.65^b^	9.68	60.60 ± 1.09^b^	18.35

#### 3.5.2 Phytotoxicity screening

No visible phytotoxic effects were observed in tea seedlings under any treatment. Both ZnO-NPs and ZnSO_4_ recorded a score of 0 on the phytotoxicity scale, similar to the controls.

#### 3.5.3 Chlorophyll content of leaves under treatments

**Figure 5:**
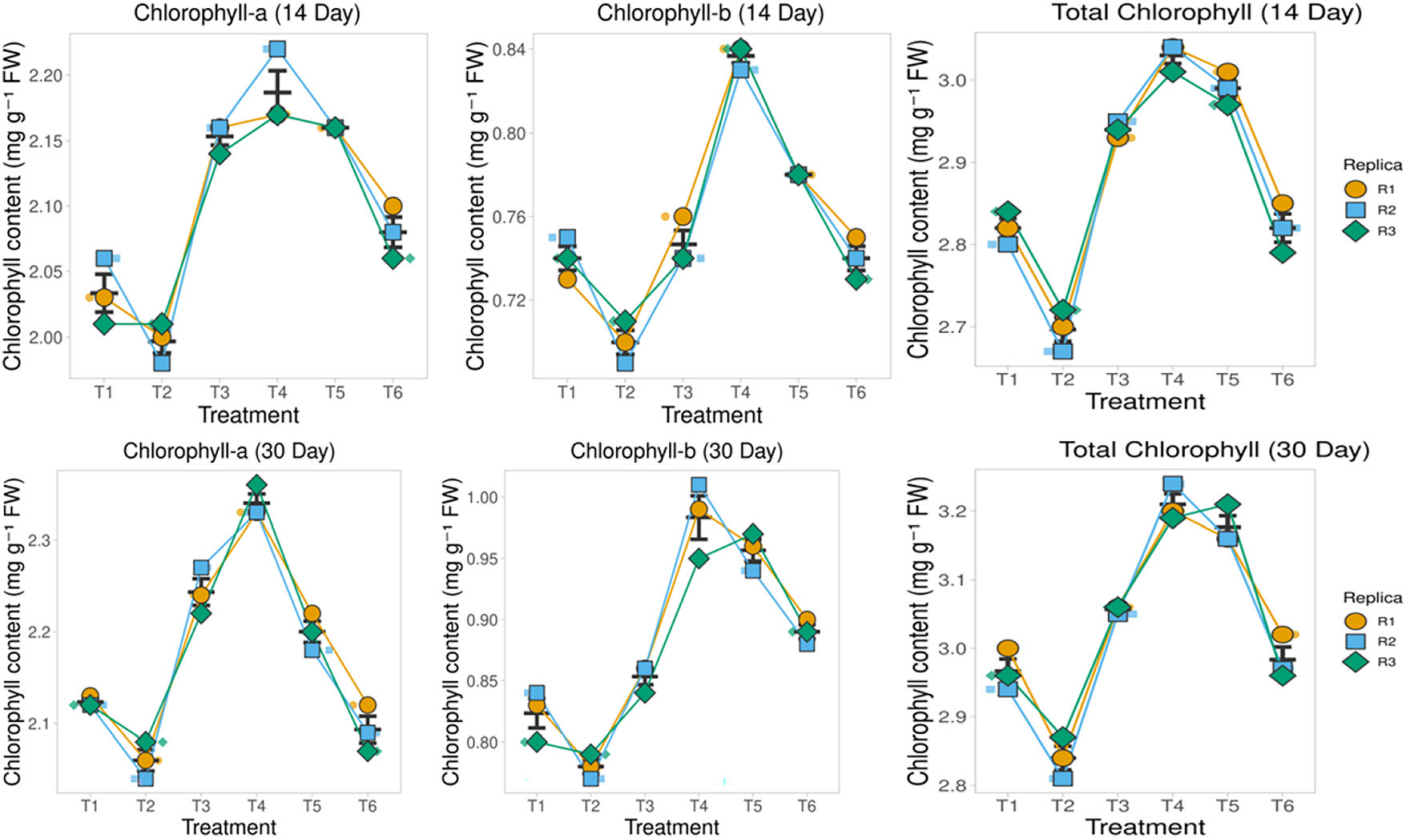
Effects of ZnO nanoparticles and ZnSO_4_·7H_2_O on chlorophyll content in tea leaves. Superplots show chlorophyll *a* (row 1), chlorophyll *b* (row 2), and total chlorophyll (row 3) across six treatments: T1, sterile control; T2, inoculated control; T3, ZnO-NP (3 mg kg^−1^); T4, ZnO-NP (6 mg kg^−1^); T5, ZnO-NP (9 mg kg^−1^); and T6, ZnSO_4_·7H_2_O (salt control). The highest accumulation was recorded under T4 and T6 (ZnSO_4_·7H_2_O), which remained lower than the equivalent nanoparticle dose.

Chlorophyll contents (Chl a, Chl b, and Total Chl) did not differ significantly among treatments at Day 0, confirming a uniform baseline. By Day 14, ZnO-NP treatments, particularly T3–T5, showed marked increases compared with the inoculated control, while T2 remained statistically similar to the control. By Day 30, the differences became more pronounced, with T4–T6 recording the highest chlorophyll concentrations and T1–T2 consistently showing the lowest. Overall, chlorophyll accumulation showed a precise dose- and time-dependent increase under ZnO-NP treatments, with a maximum response at higher concentrations and longer durations (Figure 5).

ANOVA (Treatment × Day × Chlorophyll type) showed significant main effects of treatment (F(5,72) = 383.72, p < 0.001, ω^2^ = 0.009), day (F(1,72) = 958.50, p < 0.001, ω^2^ = 0.004), and chlorophyll type (F(2,72) = 106,543.86, p < 0.001, ω^2^ = 0.984). All two-way interactions were significant – Treatment × Day (F(5,72) = 6.69, p < 0.001), Treatment × Chlorophyll type (F(10,72) = 28.57, p < 0.001), and Day × Chlorophyll type (F(2,72) = 38.06, p < 0.001). The three-way interaction was also significant (F(10,72) = 5.39, p < 0.001), indicating that treatment- and time-dependent responses differed across chlorophyll fractions.

#### 3.5.4 Leaf Zn concentration

**Figure 6:**
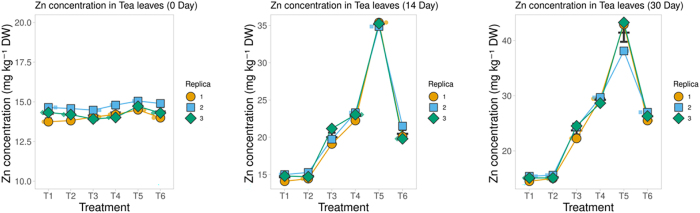
Effects of ZnO nanoparticles and ZnSO_4_·7H_2_O on Zn concentration in tea leaves. Superplots show Zn concentration (mg kg^−1^ DW) at 0 days (row 1), 14 days (row 2), and 30 days (row 3) across six treatments: T1, sterile control; T2, inoculated control; T3, ZnO-NP (3 mg kg^−1^); T4, ZnO-NP (6 mg kg^−1^); T5, ZnO-NP (9 mg kg^−1^); and T6, ZnSO_4_·7H_2_O (salt control). The highest accumulation was recorded under T5, followed by T4 and T3, while T6 (ZnSO_4_·7H_2_O) remained lower than the equivalent nanoparticle dose.

Leaf Zn concentrations in tea leaves exhibited a clear treatment- and time-dependent pattern (Figure 6). The sterile (T1) and inoculated (T2) controls maintained consistently low Zn levels throughout the experiment. Application of ZnO nanoparticles enhanced Zn accumulation in a dose-dependent manner, with the T5 producing the highest leaf Zn concentrations, followed by the T4 and T3 treatments. The ZnSO_4_·7H_2_O control (T6) also improved Zn uptake compared with controls, though its effect was lower than that of the equivalent nanoparticle dose. Across all treatments, Zn concentrations increased progressively over time, with the most substantial gains observed in the higher NP treatments (T4–T5) (Figure 6).

A two-way ANOVA (Treatment × Day) showed strong effects of treatment (F(5,36) = 377.5, p < 0.001, ω^2^ = 0.447), day (F(2,36) = 664.1, p < 0.001, ω^2^ = 0.315), and their interaction (F(10,36) = 95.5, p < 0.001, ω^2^ = 0.225), indicating that Zn accumulation varied significantly among treatments and over time. Kruskal–Wallis validation confirmed no differences at Day 0 (H = 4.62, p = 0.46), but significant treatment effects at Day 14 (H = 15.78, p = 0.007) and Day 30 (H = 16.15, p = 0.006). Tukey’s HSD further showed that higher ZnO-NP doses (T4, T5) and ZnSO_4_ (T6) differed substantially from the controls (T1, T2) and the lowest NP dose (T3), with the strongest separation occurring at later sampling intervals.

### 3.6 In vitro *Fusarium* suppression assays and SEM morphometrics

**Figure 7:**
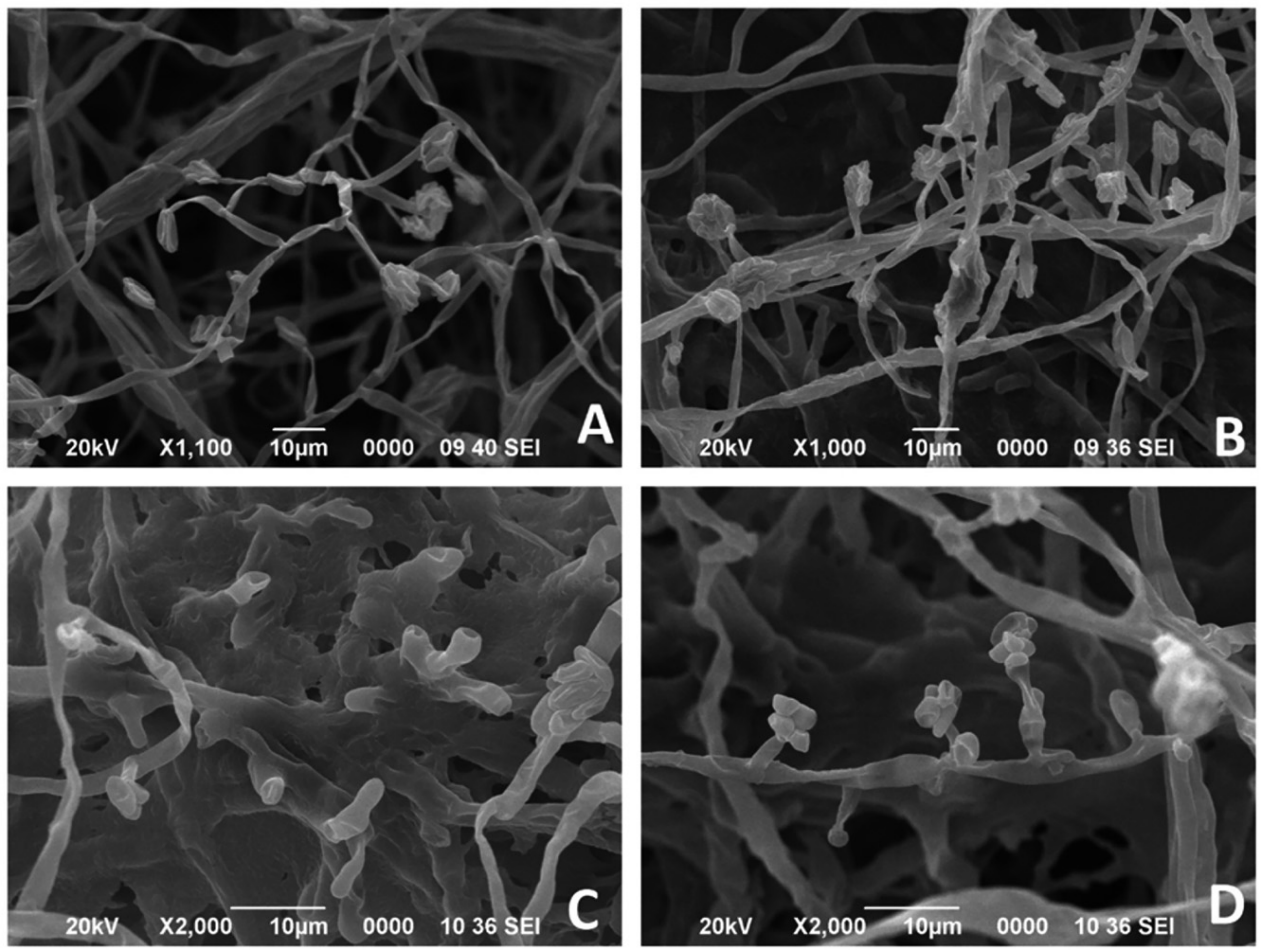
(A) Untreated control showing smooth, cylindrical hyphae with intact conidia (×1,100). (B) ZnO-NP treatment at 300 mg L^−1^ (≈300 ppm suspension) showing partial hyphal distortion and reduced conidial integrity (×1,000). (C, D) ZnO-NP treatment at 450 mg L^−1^ (≈450 ppm suspension) showing collapsed, wrinkled hyphae and severely deformed conidia (×2,000). Scale bar = 10 µm.

At 150, 300, and 450 mg L^−1^, SEM micrographs showed similar patterns of hyphal wall collapse; therefore, only the 450 mg L^−1^ treatment is presented in Figure 7. Morphometric analysis revealed apparent ultrastructural differences between treated and control samples. Control hyphae and conidia exhibited smooth walls, cylindrical outlines, and uniform ellipsoidal shapes (circularity ≈ 0.40; aspect ratio ≈ 2.2). In contrast, ZnO-NP exposure induced irregular swelling, fragmentation, and loss of uniformity, with reduced circularity (≈0.36) and lower solidity, consistent with wall collapse and increased porosity. Aspect ratio values remained broadly similar (∼2.1–2.2), though greater variability indicated non-uniform deformation. Replicate-based means reduced inflated variance compared with pooled data (Table S6), and the residual variation reflected natural heterogeneity of the fungal population rather than measurement error. One-way ANOVA confirmed significant reductions in circularity and solidity (p ≤ 0.05), whereas aspect ratio did not differ significantly.

### 3.7 Microbial biomass carbon (MBC) response to ZnO nanoparticles

Application of ZnO nanoparticles significantly influenced MBC in a precise dose- and time-dependent manner. The strongest suppression was observed at T5, which consistently recorded the lowest MBC values. Moderate declines occurred under T4 and T6, while T3 remained closer to the control (T1).

Over time, MBC declined progressively from Day 0 to Day 30, with sharper reductions at higher ZnO-NP and ZnSO_4_ (T6) doses, underscoring the cumulative nature of suppression.

A two-way ANOVA (Treatment × Day) showed significant effects of treatment (F(4,75) = 29.39, p < 0.001, ω^2^ = 0.345), day (F(2,75) = 56.28, p < 0.001, ω^2^ = 0.336), and their interaction (F(8,75) = 2.84, p = 0.008, ω^2^ = 0.045). Tukey’s HSD confirmed that MBC suppression was greatest at the highest ZnO-NP dose (T5), with the strongest reduction observed at Day 30 (Table 5).

**Table 5:** Microbial biomass carbon (MBC) of tea seedlings under ZnO nanoparticle treatments at Day 0, 14, and 30. Values are mean ± SE (*n* = 6). ΔMBC (%) represents the relative change between Day 30 and Day 0. Different superscript letters within a column indicate significant differences according to Tukey’s HSD (p ≤ 0.05).

Treatment	Day 0 (mg C kg^−1^)	Day 14 (mg C kg^−1^)	Day 30 (mg C kg^−1^)	ΔMBC (%)
T1	298.5 ± 1.6a	286.7 ± 5.2a	266.0 ± 3.8a	−10.9
T3	287.1 ± 3.0a	282.0 ± 5.8a	265.3 ± 2.5a	−7.6
T4	288.6 ± 2.8a	273.0 ± 5.7a	253.4 ± 10.4a	−12.2
T5	275.5 ± 7.8b	227.5 ± 3.0b	224.2 ± 2.1b	−18.6
T6	288.1 ± 2.1a	275.7 ± 3.6a	257.0 ± 7.9a	−10.8

## 4 Discussion

### 4.1 Antifungal efficacy and dose optimization

This study demonstrates that ZnO-NPs strongly suppressed *F. solani* compared with ZnSO_4_, confirming antifungal activity beyond the effects of Zn^2+^ ions alone. The dual-function role of ZnO-NPs is attributable to both their direct antifungal properties and their capacity to improve plant Zn nutrition. Accordingly, the reduction in *F. solani* can be explained by both (i) direct toxic effects of ZnO-NPs and (ii) indirect plant-mediated mechanisms. Direct activity is supported by *in vitro* toxicity (EC_50_ ≈ 310 mg L^−1^), SEM-documented hyphal/conidial deformation, and reduced rhizosphere CFU counts, consistent with mechanisms reported for ZnO-NPs such as particle–pathogen contact, disruption of membrane/cell wall integrity, ROS-associated damage, and interference with fungal metal homeostasis in other *Fusarium* pathosystems [15], 27]. Indirectly, the increases in DTPA-Zn, foliar Zn, and chlorophyll indicate improved Zn nutrition, which can enhance basal defense capacity through antioxidant and defense-related enzyme function [28], 29]. In addition, ISR-like responses have been reported under ZnO-NP exposure (e.g., elevated phenolics, enhanced antioxidant enzymes, and defense pathway activation) [30], [31], [32]. However, because we did not quantify defense hormones/genes or molecular markers, ISR cannot be confirmed in the present study. The specific molecular pathways underlying the observed *F. solani* hyphal/conidial deformations could not be confirmed, as gene expression or transcriptomic analyses were not performed at this time. However, our results provide stronger direct evidence for antifungal toxicity as a primary mechanism. Although molecular defense markers were not measured, these combined effects align with the observed 18–55 % reduction in disease severity.

### 4.2 Soil interactions, dissolution dynamics, and long-term fate of zinc from nanoparticle and conventional sources

Zn supplied as ZnO-NPs differs from ZnSO_4_·7H_2_O in dissolution kinetics and soil interactions. ZnSO_4_ dissociates rapidly, causing an immediate rise in soluble Zn^2+^, whereas ZnO-NPs dissolve more gradually, size- and pH-dependently, and may aggregate or become surface-coated, thereby moderating Zn^2+^ release [33], 34]. In the acidic tea soil (pH 5.43–5.49), ZnO dissolution is favored, but Zn^2+^ availability is buffered by sorption/complexation on organic matter and Fe/Al oxides and clays [35]. The increase in DTPA-extractable Zn (0.39–0.84 mg kg^−1^) indicates sustained Zn supply, and the negative pH–Zn relationship (r = −0.46 to −0.72) is consistent with higher Zn solubility at lower pH. At the same time, stable EC suggests little change in ionic strength. This pattern indicates sustained Zn supply without evidence of excessive leaching or ionic loading at the tested rates.

Evidence from soil aging studies indicates that ZnO-NPs introduced into soils undergo partial dissolution and transformation, shifting Zn into secondary species (e.g., Zn-phosphates, Zn (OH)_2_, and Zn–Al layered double hydroxides) [36]. Because dissolution releases Zn^2+^, repeated applications, especially in acidic soils, can increase total and extractable Zn over time [37]. However, sorption to organic matter/mineral surfaces and precipitation into sparingly soluble phases can limit mobility, depending on soil chemistry. In our study, the moderate DTPA-Zn increase and stable soil EC suggest no abrupt ionic loading at the tested doses, but longer-duration monitoring of total/available Zn and biological indicators is needed to assess accumulation risk.

ZnO nanoparticles can influence nutrient availability mainly through dissolution to Zn^2+^, which can compete for sorption/complexation sites on Fe/Mn oxides, clays, and organic matter and may contribute to Fe/Mn–Zn antagonism during uptake [38]. Zinc inputs may also alter P availability via Zn-phosphate precipitation and adsorption reactions, and by influencing enzyme-mediated P cycling in low-P soils [39], 40]. Reported field evidence suggests that low-dose ZnO-NPs can improve Zn acquisition without materially disrupting *N* and P uptake, indicating soil- and dose-dependent outcomes [41].

### 4.3 Microbial stability, phytotoxicity, and ecological implications of ZnO-NPs

In our study, the potential microbial toxicity of ZnO-NPs was evaluated in an auxiliary non-sterile pot experiment using field soil to maintain an intact native microbial community. Microbial biomass carbon (MBC) was used as a functional indicator of microbial activity. Although microbial community composition was not assessed, the results showed that lower ZnO-NP doses caused only modest declines in MBC (7–12 % at 3–6 mg kg^−1^), suggesting minimal microbial stress at agronomically relevant concentrations. In contrast, the highest application rate (9 mg kg^−1^) resulted in a more pronounced reduction in MBC (−18.6 %), indicating dose-dependent microbial sensitivity. Similar dose-dependent responses have been reported in other soil systems, where low ZnO-NP applications (≤5–6 mg kg^−1^) generally exert minimal microbial disturbance. In contrast, higher concentrations lead to measurable inhibition of microbial functions. Singh et al. [42] reported that 5 mg kg^−1^ ZnO-NPs preserved microbial biomass and bacterial populations while enhancing Zn nutrition in wheat, supporting the view that low mg kg^−1^ applications are generally agronomically safe. Similarly, Shen et al. [43] observed reductions in dehydrogenase and related enzyme activities at 5–10 mg kg^−1^, with more potent inhibition at 50–100 mg kg^−1^, demonstrating that microbial stress may begin even at moderate doses, depending on soil conditions, consistent with our experiment.

Overall, the consistency between our MBC and CFU results and previously published studies indicates that soil microbial communities generally withstand low-to-moderate ZnO-NP additions. In contrast, progressively higher concentrations tend to suppress microbial activity more markedly. Collectively, these findings indicate that microbial communities tolerate low-to-moderate applications but may approach ecotoxicological thresholds under higher doses.

Evidence suggests that low, agronomic doses of ZnO-NPs cause microbial responses broadly comparable to ZnSO_4_, including no consistent reduction in beneficial bacteria functions or arbuscular mycorrhizal colonization at low mg kg^−1^ ranges [44], 45]. In contrast, higher doses and/or repeated applications can suppress enzyme activity and microbial biomass [43].

In this study, MBC and CFU showed controlled, dose-dependent responses rather than collapse. Still, community-level shifts in beneficial taxa or mycorrhizae would require targeted assays (e.g., AMF colonization and sequencing) [39].

No phytotoxic symptoms were observed at the tested doses (3–9 mg kg^−1^), and chlorophyll and visual growth traits remained stable or improved. However, published evidence indicates that phytotoxicity and ecotoxicity can emerge at higher doses and/or with prolonged or repeated exposure (e.g., growth inhibition, oxidative stress, reduced microbial biomass/enzyme activities), and risk is therefore dose-, time-, and soil-dependent [45], [46], [47]. The use of nanomaterials in soil systems raises several environmental concerns, particularly the potential for zinc accumulation and the persistence of nanoparticles over time. Evidence from agronomic studies indicates that low ZnO-NP applications (≤10 mg kg^−1^) generally act as a supplemental zinc source rather than as a toxic input, enhancing plant performance and beneficial soil–plant interactions [42]. In contrast, elevated concentrations have been associated with disruptions to earthworm physiology [48], shifts in plant–soil dynamics [49], and reductions in critical soil enzyme activities. Long-term assessments, including those by Kwak et al. [47], show that ZnO-NP treatments at 50–500 mg kg^−1^ can alter enzymes such as acid phosphatase and urease, suggesting possible consequences for phosphorus and nitrogen cycling within soil ecosystems.

### 4.4 Field relevance, limitations, and future directions

The present study was restricted to a short-term (30-day) greenhouse trial designed to study the initial dose–response behavior of the soil–plant–pathogen system during the establishment period. This controlled setup allowed us to understand nanoparticle effects on *F. solani* suppression, Zn uptake, and early physiological responses without interference from seasonal or field-level variability.

Field performance may differ from greenhouse conditions because soil heterogeneity, rainfall, UV exposure, and complex microbial networks can alter ZnO-NP dissolution, transport, and persistence. Reported field studies show benefits for Zn uptake, yield, and stress tolerance, but responses vary with soil chemistry and environmental conditions [44], 45]. Evidence from tea phyllosphere work [50] and *in vivo Fusarium* suppression in eggplant [23] suggests protective effects are possible under biologically diverse conditions. However, multi-location field trials are needed to confirm consistency.

## 5 Conclusions

Zinc oxide nanoparticles (ZnO-NPs) acted as dual-function soil amendments in tea, enhancing zinc bioavailability while suppressing *F. solani* dieback under greenhouse conditions. The intermediate dose (6 mg kg^−1^) provided the most favorable balance between disease control and microbial stability, highlighting the importance of dose optimization. Compared with conventional ZnSO_4_, ZnO-NPs maintained higher and more persistent levels of bioavailable Zn in acidic soils, leading to improved uptake efficiency in tea seedlings. These results provide preliminary greenhouse-scale evidence that ZnO-NPs can serve as a nano-enabled approach for integrated nutrient and disease management in tea. Validation under multi-season and field-scale trials will be essential to confirm efficacy, ecological safety, and agronomic relevance.

## Supplementary Material

Supplementary Material Details
