# Supplementary material for: Zinc oxide nanoparticles enable sustainable disease management in tea by dual nutrient and antifungal action
Source: Open Life Sci. 2026 Feb 3;21(1):20251260. doi: 10.1515/biol-2025-1260 (PMC12915709; doi:10.1515/biol-2025-1260)
Supplement: Supplementary file 1 — Supplementary Material Details [file j_biol-2025-1260_suppl_001.docx]

**Zinc Oxide nanoparticles enable sustainable disease management in tea by dual nutrient and antifungal action**

# Supplementary Methods 1

## Soil Preparation and Analysis

**Sterilization:** Soil used for pathogen-inoculated pot assays was sterilized by autoclaving at 121 °C and 15 psi for 60 minutes on two consecutive days.

**Soil Suspension:** Baseline soil physicochemical properties were measured using a 1:5 soil-to-water suspension ratio.

**DTPA-Extractable Zn:** Ten grams of soil were extracted with 20 mL of 0.005 M diethylenetriaminepentaacetic acid (DTPA) solution (pH 7.3). The extract was filtered through Whatman No. 42 filter paper and analyzed for Zn concentration using an atomic absorption spectrophotometer (PerkinElmer AAnalyst 400).

**Soil pH and EC:** Measurements were conducted with a Mettler Toledo Five Easy Plus pH/EC meter.

**Soil Texture:** Texture was classified using the hydrometer method with a Humboldt Model 152H Hydrometer (USA).

# Supplementary Methods 2

# ZnO Nanoparticle Characterization

## X-ray Diffraction (XRD)

The crystalline structure of ZnO-NPs was determined using a Bruker AXS D8 FOCUS diffractometer with Cu Kα radiation (λ = 1.5406 Å), scanning from 20–80° (2θ). Diffraction peaks were indexed to the hexagonal wurtzite phase (JCPDS card no. 36-1451). Crystallite size was estimated by the Scherrer equation: D = Kλ / (β cosθ), where D = crystallite size, K = shape factor (0.9), λ = X-ray wavelength, β = full-width half maximum (FWHM), and θ = Bragg angle. Micro-strain (ε) was calculated using the Williamson–Hall method: βcosθ = (Kλ / D) + 4εsinθ.

## Fourier Transform Infrared Spectroscopy (FTIR)

Surface functional groups were identified using FTIR (PerkinElmer Spectrum 100) operated at 4 cm⁻¹ resolution, 32 scans per sample over 400–4000 cm⁻¹. KBr pellets were prepared by mixing finely ground ZnO-NPs with spectroscopic-grade KBr in a ratio of 1:100. Bands were assigned using standard vibrational modes.

## Field Emission Scanning Electron Microscopy (FESEM)

Morphology and particle size distribution were examined with FESEM (JEOL, Japan) at 5 kV accelerating voltage and 50,000× magnification. Samples were sputter-coated with gold prior to imaging. Particle dimensions were quantified from micrographs using ImageJ software, with at least 150 particles measured per field.

## Energy-Dispersive X-ray Spectroscopy (EDS)

## The elemental composition of the synthesized sample was analyzed by Energy-Dispersive X-ray Spectroscopy (EDS) attached to a Scanning Electron Microscope (JEOL, Japan) The analysis was carried out under high vacuum with an accelerating voltage of 20 kV. Standard reference materials (SiO₂ for O and pure Zn metal for Zn) were used for calibration. The EDS spectrum was collected over the energy range of 0–12 keV, and elemental quantification was performed using ZAF correction procedures, normalized to 100%. Multiple iterations were used to refine peak identification and quantification.

## Zeta Potential (ζ-potential)

The ζ- potential (ZP) of ZnO-NPs nanoparticle suspension (800 ppm) was determined using a Zetasizer Nano ZS (Malvern Instruments Ltd., UK) at 25 °C. Samples were dispersed in double-distilled water by ultrasonication (15 min) prior to measurement to ensure homogeneity. Measurements were performed in a clear disposable zeta cell. Instrument parameters included dispersant refractive index (1.330), dielectric constant (78.5), and viscosity (0.8872 cP). Each sample was analyzed in triplicate, and electrophoretic mobility (µm cm/Vs) was recorded simultaneously. The conductivity (mS/cm) and wall zeta potential (mV) were also obtained during measurement and results were expressed as mean ± SD

## Table S1. Details of ZnO-NP and ZnSO₄·7H₂O treatments applied in the greenhouse pot experiment.

| Treatment | Solution concentration (mg L⁻¹, ppm) | ZnO applied per 100 mL drench (mg) | Soil mass per pot (kg) | Effective soil dose (mg ZnO kg⁻¹ soil) | Elemental Zn delivered (mg Zn kg⁻¹ soil) |
| --- | --- | --- | --- | --- | --- |
| (T1) Sterile control (DW) | - | - | 5 | - | - |
| (T2) *F. solani* control | - | - | 5 | - | - |
| (T3) ZnO-NPs + *F. solani* | 150 | 15 | 5 | 3 | 2.41 |
| (T4) ZnO-NPs + *F. solani* | 300 | 30 | 5 | 6 | 4.82 |
| (T5) ZnO-NPs + *F. solani* | 450 | 45 | 5 | 9 | 7.23 |
| (T6) ZnSO₄·7H₂O + *F. solani* | Equivalent Zn to NP-High | 31.8 | 5 | Equivalent (matched to T5) | 7.23 |

- Notes:
- ppm = mg L⁻¹ in drench solution.
- Each pot contained 5 kg of soil; soil doses were calculated based on this weight.
- Elemental Zn fraction in ZnO = 65.38 / 81.38 (≈0.803).
- Elemental Zn fraction in ZnSO₄·7H₂O = 65.38 / 287.55 (≈0.227).
- For the ZnSO₄·7H₂O treatment, the applied dose was adjusted to match the elemental Zn content of the highest ZnO-NP treatment (T5).
- At transplanting, each pot received 100 mL of the respective suspension as a soil drench.
- Concentration ranges were selected based on preliminary antifungal bioassays against *F. solani* (EC₅₀ ≈ 310 mg L⁻¹).

**Table S2. EC₅₀ values from dose–response screening of *Fusarium solani* with ZnO-NPs.**

In vitro antifungal activity of ZnO-NPs against *Fusarium solani*. The table shows percent inhibition (mean ± SD, n = 3) at different concentrations. The EC₅₀ value was estimated 310 µg mL⁻¹

| **Concentration (µg mL⁻¹)** | **% Inhibition ± SD** |
| --- | --- |
| 100 | 27.8 ± 0.30 |
| 200 | 44.2 ± 0.95 |
| 400 | 52.5 ± 1.40 |
| 600 | 71.6 ± 2.60 |
| 800 | 85.9 ± 1.35 |
| 1000 | 86.1 ± 2.70 |
| 1200 | 82.0 ± 2.30 |

# Supplementary Methods 3

## Molecular Identification and Phylogenetic Analysis

Genomic DNA was extracted from fresh Fusarium solani mycelium using a CTAB–phenol–chloroform protocol with minor modifications. The internal transcribed spacer (ITS) region of rDNA was amplified using universal primers (ITS1 and ITS4). PCR products (~550 bp) were purified and sequenced on an ABI 3130 Genetic Analyzer (Applied Biosystems). Raw chromatograms were edited and assembled using SeqScape™ v5.2.

The ITS sequence of the isolate DSP-1 (~593 bp) was deposited in GenBank under accession number PV171106.1. Sequence identity was confirmed by BLASTN search against the NCBI database.

For phylogenetic analysis, ITS sequences were aligned with representative Fusarium spp. and selected tea pathogens as outgroups. Alignments included 674 positions (1st, 2nd, and 3rd codon positions). The evolutionary history was inferred using the Maximum Likelihood (ML) method in MEGA v12. The initial tree for heuristic search was generated using Neighbor-Joining and Maximum Parsimony algorithms applied to pairwise distances computed with the p-distance model. Node support was assessed with 1,000 bootstrap replicates, and branches reproduced in fewer than 50% of replicates were collapsed. Analyses were performed using four parallel computing threads.

## Table S3. Disease severity index (DSI) scoring for tea seedlings.

Disease severity was scored on individual leaves using a 0–5 scale. Scores were assigned based on the percentage of infected leaf area, adapted from Sarmah et al. (2020). For each pot, leaf scores were converted to a pot-level DSI% using the formula provided in the main text.

| **Score** | **Descriptions** |
| --- | --- |
| 0 | No visible symptoms |
| 1 | ≈1% of leaf area infected |
| 2 | 2–10% of leaf area infected |
| 3 | 11–50% of leaf area infected |
| 4 | 51–75% of leaf area infected |
| 5 | >75% of leaf area infected |

## Table S4. Phytotoxicity grading system for tea seedlings.

Phytotoxicity grading system used for ZnO-NP treatments in tea seedlings. Seedling injury was scored on a 0–10 scale based on the percentage of observed damage, ranging from 0 (no visible effect) to 10 (91–100% injury). Descriptive symptoms included tip burn, necrosis, wilting, vein clearing, epinasty, and hyponasty.

| **Percentage Injury** | **Grade (0–10 scale)** |
| --- | --- |
| 0% | 0 |
| 0–10% | 1 |
| 11–20% | 2 |
| 21–30% | 3 |
| 31–40% | 4 |
| 41–50% | 5 |
| 51–60% | 6 |
| 61–70% | 7 |
| 71–80% | 8 |
| 81–90% | 9 |
| 91–100% | 10 |

Note: The DSI was adapted from Sarmah et al. (2020). The phytotoxicity scale follows Bassanezi et al. (2011).

## Supplementary Table S5. Details of chlorophyll estimation and Zn analysis in tea seedlings.

This table summarizes the technical details of chlorophyll extraction and foliar Zn determination, which were condensed in the main Methods section.

| Parameter | Details |
| --- | --- |
| Instrument (UV–Vis) | Shimadzu UV-2600i (Japan) |
| Extraction solvent | 96% ethanol |
| Leaf tissue weight | ≈0.2 g fresh leaf tissue |
| Extraction volume | 60 mL ethanol, incubated in dark at room temperature |
| Absorbance wavelengths | 665 and 649 nm |
|  |  |
| Instrument (AAS) | PerkinElmer AAnalyst 400 |
| Sample preparation (Zn) | Leaves oven-dried at 65 °C to constant weight, finely ground. ≈0.2 g digested with HNO₃:HClO₄ (3:1 v/v), diluted to 25 mL, filtered (Whatman No. 42). |
| Output expression | Leaf Zn concentration expressed as mg Zn kg⁻¹ dry weight (DW) |

# Supplementary Methods 3

## Supplementary Information for In vitro Fusarium suppression assays and SEM observations

This section provides detailed technical information for ZnO-NP antifungal assays against Fusarium solani and corresponding SEM morphometric analyses.

### Experimental Details

Culture medium: Potato dextrose broth (HiMedia, India).

ZnO-NP suspension: Prepared in sterile distilled water containing 0.01% Tween-80 and probe-sonicated for 10 min in an ice bath before application.

SEM: JEOL JSM-6390LV scanning electron microscope operated at 5 kV with a secondary electron detector.

Probit analysis: Performed using OriginPro 2018.

Sample preparation: Mycelia and conidia were fixed in 2.5% glutaraldehyde, washed in phosphate buffer, and dehydrated through a graded ethanol series (30%, 50%, 70%, 90%, 100%). Samples were air-dried, gold-coated, and mounted prior to SEM imaging.

## Supplementary Table S6. Morphometric measurements of *Fusarium solani* under ZnO-NP treatments.

For morphometric analyses, at least 100 hyphae and 200 conidia were measured per treatment across three independent culture replicates. Measurements were averaged within each replicate to generate a single replicate mean for each morphometric parameter (e.g., area, perimeter, circularity). These replicate means (n = 3 per treatment) were then subjected to one-way ANOVA followed by Tukey’s HSD.

Morphometric parameters of *Fusarium solani* under control and ZnO-NP treatment (9 mg L⁻¹). Values represent mean ± SE of replicate means (n = 3 independent cultures). Different superscript letters within a row indicate significant differences according to Tukey’s HSD test (p ≤ 0.05). Shared letters indicate non-significant differences.

| Parameter | Control (Mean ± SE) | ZnO-NP Treated (Mean ± SE) | p-value (ANOVA) | Tukey’s Grouping |
| --- | --- | --- | --- | --- |
| Area (µm²) | 116.6 ± 22.9ᵇ | 181.4 ± 31.5ᵃ | <0.05 | a, b |
| Perimeter (µm) | 56.0 ± 6.3ᵇ | 76.3 ± 9.1ᵃ | <0.05 | a, b |
| Feret (µm) | 16.0 ± 1.2ᵇ | 17.2 ± 1.4ᵃ | <0.05 | a, b |
| MinFeret (µm) | 8.2 ± 0.7ᵇ | 9.6 ± 0.8ᵃ | <0.05 | a, b |
| Circularity | 0.396 ± 0.014ᵃ | 0.359 ± 0.016ᵇ | <0.05 | a, b |
| Aspect Ratio | 2.17 ± 0.11ᵃ | 2.11 ± 0.10ᵃ | p = 0.41 | a |
| Solidity | 0.823 ± 0.007ᵃ | 0.780 ± 0.009ᵇ | <0.05 | a, b |

**Table S7** *Zeta potential (ZP), electrophoretic mobility, conductivity, and wall zeta potential of ZnO-NP suspension (800 ppm) at 25 °C (n = 3).*

| Record | Type | Sample Name | T | ZP | Mob | Cond | Wall Zeta Potential | |
| --- | --- | --- | --- | --- | --- | --- | --- | --- |
|  |  |  | °C | mV | µmcm/Vs | mS/cm | mV |  |
| 1 | Zeta | 800ppm 1 | 25 | 17.7 | 1.385 | 0.097 | -10.4 |  |
| 2 | Zeta | 800ppm 2 | 25 | 19.3 | 1.51 | 0.0977 | -12 |  |
| 3 | Zeta | 800ppm 3 | 25 | 18.5 | 1.448 | 0.0968 | -10.6 |  |

## References

1. Walkley, A., & Black, I.A. (1934). An examination of the Degtjareff method for determining soil organic matter, and a proposed modification of the chromic acid titration method. Soil Science, 37, 29–38.
2. Black, C.A., Evans, D.D., Ensminger, L.E., White, J.L., Clark, F.E., & Dinauer, R.C. (2016). Methods of Soil Analysis. Part 2: Chemical and Microbiological Properties. American Society of Agronomy, Madison, WI.
3. Martens, D.C., & Lindsay, W.L. (2018). Testing soils for copper, iron, manganese, and zinc. In: Walsh, L.M., Beaton, J.D. (Eds.), Soil Testing and Plant Analysis. Soil Science Society of America, Madison, WI.
4. Bouyoucos, G.J. (1962). Hydrometer method improved for making particle size analyses of soils. Agronomy Journal, 54, 464–465.
